# Differences in the presentation of COVID-19-related psychosocial stress and general psychological distress and the relation between the number of care days and these symptoms among Japanese ward staff working exclusively with COVID-19 and support staff

**DOI:** 10.1186/s13030-023-00272-7

**Published:** 2023-04-28

**Authors:** Tomoe Nishihara, Kazufumi Yoshihara, Ayako Ohashi, Mika Kuroiwa, Nobuyuki Sudo

**Affiliations:** 1 Department of Psychosomatic Medicine, National Hospital Organization Fukuoka Higashi Medical Center, Fukuoka, Japan; 2 Department of Psychosomatic Medicine, Graduate School of Medical Sciences, Kyushu University, 3-1-1 Maidashi, Higashi-Ku, Fukuoka-City, 812-8582 Japan; 3 Division of Health Promotion and Development, Center for Health Sciences and Counseling, Kyushu University, Fukuoka, Japan; 4 Department of Psychiatry, National Hospital Organization Fukuoka Higashi Medical Center, Fukuoka, Japan; 5 Department of Neuropsychiatry, Graduate School of Medical Sciences, Kyushu University, Fukuoka, Japan; 6 Department of Clinical Research Center, National Hospital Organization Fukuoka Higashi Medical Center, Fukuoka, Japan

**Keywords:** COVID-19, Health care worker, Support staff, Occupational stress, General psychological distress

## Abstract

**Background:**

Psychological distress has been frequently observed in frontline healthcare workers under stress during the coronavirus disease 2019 (COVID-19) epidemic; however, it is unclear if there are differences in the stress and symptoms experienced by staff members who work exclusively in a COVID-19 ward and support staff temporarily deployed to a COVID-19 ward. The present study investigated psychosocial stress specific to the care for patients with COVID-19 and psychological distress among ward staff working exclusively with COVID-19 and temporary support staff.

**Methods:**

The participants were full-time nurses and doctors working in COVID-19 wards or the ICU who provided face-to-face care to patients with COVID-19 during the COVID-19 outbreak in February of 2021. The data of 67 staff members (21 exclusively working with Covid-19 patients (group A) and 46 in the temporary support group (group B)) was available for study. Psychosocial stress specific to healthcare professionals during this COVID-19 outbreak (Tokyo Metropolitan Distress Scale for Pandemic [TMDP]) and general psychological distress (K6) were assessed.

**Results:**

The K6 score was significantly lower in group B than in group A (*p* = .006), but no significant difference was found in the total score of TMDP or its subscales. Positive correlations were found between TMDP and K6 for group B (*p* = .011), as was the number of days of care on TMDP-social (rs = .456, *p* = .001).

**Conclusion:**

Even though support staff members experienced lower psychological distress than staff working exclusively with COVID-19, COVID-19-related psychosocial stress specific to HCWs was comparable. The support staff also presented psychological distress associated with psychosocial stress specific to healthcare professionals during this COVID-19 outbreak, and the COVID-19-related social stress was enhanced as the number of working days increased. Our results show that all staff, not only those working exclusively with COVID-19 patients but also other support staff should be provided with care focusing on COVID-19-related psychosocial occupational stress.

**Supplementary Information:**

The online version contains supplementary material available at 10.1186/s13030-023-00272-7.

## Background

A large body of evidence indicates mental health problems among healthcare workers (HCWs) engaged in caring for coronavirus disease 2019 (COVID-19) patients. A systematic review of mental health among HCWs during the COVID-19 epidemic showed that a significant proportion presented with depression, anxiety, and sleep disturbances during the period of the epidemic [1]. Anxiety and depressive symptoms have also been reported to be highly prevalent in Japanese HCWs [2]. It has long been known that depression and anxiety are associated with somatic symptoms [3]. During the COVID-19 outbreak, healthcare workers were more likely to have somatic symptoms than before the outbreak, such as pain and insomnia, which have been shown to be significantly associated with depression and anxiety [4]. Research has also shown that occupational stress, such as from an increased workload, is greater in healthcare workers treating COVID-19 patients and that these occupational stress factors are associated with psychological distress [5]. Furthermore, our previous study of frontline nurses found that COVID-19-related occupational stress and psychological distress were associated with increased workload and physical symptoms (particularly insomnia) during the first wave of the COVID-19 outbreak in Japan [6].

The prolonged outbreak of COVID-19 has forced most designated infectious disease hospitals to change the resourcing of frontline HCWs. To strengthen the limited capacity of the healthcare system, many facilities have dispatched staff working full-time in other wards to COVID-19 wards as support staff for temporary periods to compensate for the manpower shortage in inpatient care for COVID-19 patients. In previous reports, frontline medical staff providing face-to-face care to COVID-19 patients presented higher levels of depression, anxiety and insomnia than non-frontline staff [7]. Because both workers who care exclusively for COVID-19 patients and temporary support staff are frontline HCWs who provide patient care, they should be considered candidates for mental health support. However, no investigation has been done to determine if there are differences between the stress and mental symptoms of workers assigned exclusively to COVID-19 wards and temporary support staff. Whether or not the number of days spent caring for COVID-19 patients is associated with increased stress and/or symptom exacerbation has also not been reported. Clarification of these would make it possible to identify the differences in the nature of stress based on the working pattern of HCWs’ during prolonged outbreaks of an emerging infectious disease, which would enable us to provide effective support according to the working pattern. This information will allow us to provide a better working environment that will enable HCWs to maintain good mental health and well-being during emerging infectious disease epidemics.

## Hypothesis

Our hypotheses were that 1) COVID-19 related psychosocial stress and general psychological distress would be lower among the support staff of COVID-19 wards than among staff working exclusively there; and 2) the greater the number of days spent in the care of COVID-19 patients, the stronger COVID-19 related psychosocial stress and general psychological distress would be.

## Methods

### Study design

**Fig. 1:**
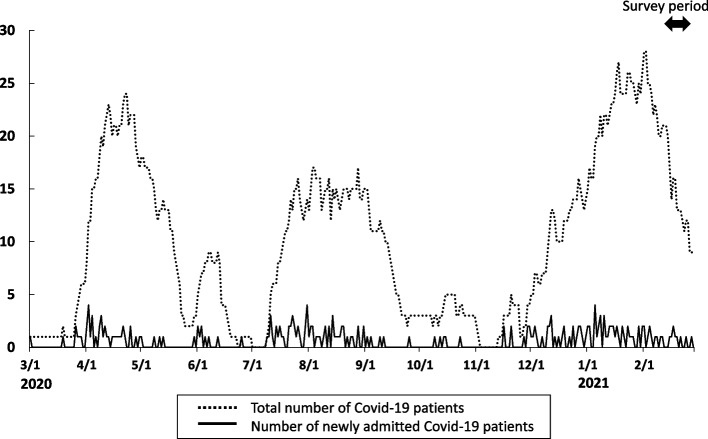
Change in the total number and number of newly admitted patients with COVID-19 at the National Hospital Organization Fukuoka-Higashi Medical Center. The peak of the third COVID-19 epidemic wave was from December 2020 to February 2021. The survey was performed from February 14–28, at the end of the peak of the third wave of COVID-19. The solid and dotted lines show the number of newly admitted patients and the total number of COVID-19 patients hospitalized, respectively

This study was a hospital survey conducted at the National Hospital Organization Fukuoka Higashi Medical Center, a designated medical institution for infectious diseases in Fukuoka, Japan. The study protocol was approved by the National Hospital Organization Fukuoka Higashi Medical Centre Clinical Research Ethics Committee (Ref. No. 2021-C-11). This study was conducted between February 15 and 28, 2021, approximately 12 months after the first admission of COVID-19 patients to our hospital during the first wave of the outbreak in Japan (Fig. 1).

### Study subjects

Frontline nurses and doctors providing face-to-face care to COVID-19 patients in the COVID-19 ward were included in the study. We included both physicians and nurses because they were not different in the amount of time they spent in daily patient care duties in a ward and wore the same personal protective equipment. Seventy-three eligible HCWs were recruited by informing them of the purpose of the occupational stress survey. Staff working full-time exclusively in COVID-19 wards were assigned to a group A, and staff previously working full-time in other wards but sent to the COVID-19 ward as support staff for limited periods during the peak period of COVID-19 outbreaks were assigned to a group B.

Participants were asked about demographic data, such as sex, occupation (doctor or nurse), history of working as a healthcare professional, number of days spent providing care for COVID-19 patients, medical history (regarding mental illness and physical symptoms), and if they lived alone. By the end of the survey period, no cluster infections had occurred and no participants were infected with COVID-19 in our hospital, so we did not inquire if they themselves had been affected by COVID-19.

### Questionnaires

Both COVID-19-related psychosocial stress specific to healthcare professionals caring for patients with COVID-19 and general psychological distress were investigated using the following self-administered questionnaires.

#### Tokyo Metropolitan Distress Scale for Pandemic (TMDP)

The Tokyo Metropolitan Distress Scale for Pandemic (TMDP) is a standardized questionnaire developed to assess pandemic-related infection concerns (psychological stress) and social stress among healthcare workers providing care for COVID-19 patients [8]. The items of TMDP are presented as Supplementary Material 1. Respondents are asked about their frequency concern during the past two weeks about nine items. The respondent answered each item using a 5-point Likert scale ranging from 0 (never) to 4 (most of the time). The cutoff value for determining the need for mental health care was 14 points.

The TMDP subscale on concern about infection (psychological stress) includes question items such as ‘COVID-19 infection of oneself’, ‘you have no control over whether you have COVID-19 or not’, ‘risk of COVID-19 patient care is unacceptable’, ‘safety of the work environment is not maintained in order to avoid being affected by COVID-19’ and ‘transmitting COVID-19 to people around you’. Another subscale of the TMDP, the social stress questions, asked: ‘people around you avoid you because of your occupation’, ‘deteriorating workplace relationships in relation to COVID-19’, ‘deteriorating family relationships in relation to COVID-19’ and ‘financial burden associated with COVID-19’.

#### General psychological distress

The six-item Kessler scale (K6) was used to assess general psychological distress, such as depression and anxiety. The items of K6 are presented as Supplementary Material 2. Each item was rated by asking participants to respond on a five-point Likert scale ranging from 0 (none of the time) to 4 (All of the time). Moderate psychological distress was defined as a total score of 5–12, while the cutoff value for severe psychological distress was 13 [9, 10].

### Statistical analysis

Statistical analysis was performed using the SPSS version 22.0 J statistical software package (IBM SPSS Statistics, Chicago, IL, USA). Epidemiological data were compared using the chi-square test. Analyses were performed on the scores of the TMDP and the subscales of the TMDP and K6. Differences between the groups were analyzed using the Mann–Whitney U test. Spearman’s correlation analysis was also performed to test for correlations between these measures. The criterion for statistical significance was set at *p* < 0.05 (two-tailed). To test the hypotheses, we used the Bonferroni correction to adjust for the increased risk of a type I error when making multiple statistical tests. *p* < 0.0125 (0.05/4) (two-tailed) was considered statistically significant.

## Results

Sixty-seven participants (17 doctors and 50 nurses) completed the survey. Their age ranged from 25 to 56 years, and 22 were male (32%) Twenty-one were placed in the exclusive group and 46 in the support group, with no significant difference in age, experience working as a medical professional, proportion of doctors and nurses, medical history, or percentage of single people living alone. The number of days spent caring for COVID-19 patients was significantly higher in group A (Table 1).

**Table 1:** Participant characteristics

Variables	Group A medical staff (*n* = 21)	Group B medical staff (*n* = 46)	Statistics	*p*-value
Sex			0.25	0.62
Male (*n* = 22, 32.8%)	6 (28.6%)	16 (34.8%)		
Female (*n* = 45, 67.2%)	15 (71.4%)	30 (65.2%)		
Age (mean ± SD)	37.8 ± 10.7	37.1 ± 9.3	477.5	0.94
Occupation			0.04	0.84
Nurse (50, 74.6%)	16 (76.2%)	34 (73.9%)		
Doctor (17, 25.4%)	5 (23.8%)	12 (26.1%)		
Length of service				
Working as HCW (months)	167.9 ± 134.7	171.3 ± 148.7	452.0	0.68
Care for COVID-19 (days)	233.0 ± 68.3	47.0 ± 30.2	26.5	< 0.01 ^**^
Previous medical history			3.8	0.05
Nothing (54, 80.6%)	14 (66.7%)	40 (85.1%)		
Any one of histories (13, 19.4%)^a^	7 (33.3%)	6 (13.0%)		
Living alone (23, 34.3%)	6 (28.6%)	17 (37.0%)	0.45	0.50

The statistics data are shown as χ2 [2] value and significance levels (^*^ *p* < 0.05, ^**^ *p* < 0.01)

^a^Medical history: a psychiatric history was reported by 2 participants in group A (9.5%); insomnia was reported by 4 participants in group A (19.0%) and 2 in group B (4.3%); and other physical symptoms were reported by 2 participants in group A (9.5%) and 1 in group B (2.2%) (multiple responses allowed)

Group A worked exclusively in Covid-19 wards and Group B temporarily

Data are presented as mean ± standard deviation or n (%)

Chi-square analysis was used to test differences in categorical variables, and the Mann–Whitney test was used to test for differences in continuous values

### Differences in TMDP and general psychological distress (K6) scores

**Fig. 2:**
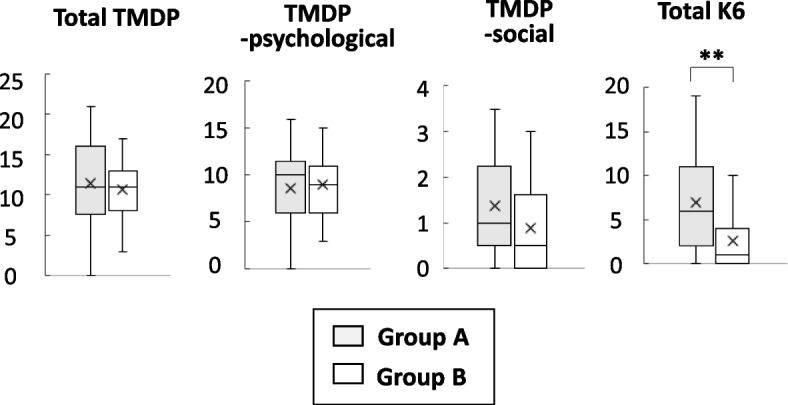
Comparison of TMDP and K6 scores of healthcare workers working exclusively (group A) or temporarily (group B) in COVID-19 wards

Figure 2 shows the total scores of TMDP and the subscales of TMDP and K6. The K6 score was significantly lower (*r* = -0.34, *p* = 0.006) for group B than for group A. No significant between group difference was found in the total score of TMDP (*r* = -0.06, *p* = 0.606) or its subscales (TMDP-psychological: *r* = -0.01, *p* = 0.908, TMDP-social: *r *= -0.22, *p* = 0.075).

### Correlations between TMDP and general psychological distress (K6) scores

A positive correlation was found between TMDP and K6 scores of group B (rs = 0.370, *p* = 0.011), but no correlation was found for group A.

### Association between the number of days caring for COVID-19 patients and TMDP or general psychological distress

To clarify the association between the number of days spent caring for COVID-19 patients and increased stress and/or symptom exacerbation, correlations between the number of days engaged in COVID-19 care and each measure were analyzed for both groups **(**Table 2**)**. A positive correlation was found in group B between the number of days and TMDP-social score (rs = 0.456, *p* = 0.001). For group A, no significant correlation was found between the number of days and the TMDP or the general psychological distress scores.

**Table 2:** Correlations between engagement days and the other variables

	Engagement days	
	***Group A (n* = *21)***	***Group B (n* = *46)***
TMDP	rs = .065	rs = .296
	*p* = .778	*p* = .046
TMDP- psychological	rs = -.107	rs = -.002
	*p* = .643	*p* = .989
TMDP-social	rs = .246	rs = .456^**^
	*p* = .283	***P* < .001**
K6	rs = .350	rs = .010
	*p* = .119	*p* = .948

The data are shown as R-values (correlation coefficient; rs) and significance levels (*p < 0.0125, ** *p* < 0.0025)

Group A worked exclusively in Covid-19 wards and Group B temporarily

## Discussion

The present study showed that temporary support staff temporarily dispatched from other wards to COVID-19 wards experienced less psychological distress than staff working exclusively in these wards, even though the stress specific to COVID-19 care was comparable. Also, they presented general psychological distress associated with COVID-19-related psychosocial stress. The COVID-19-related social stress specific to these HCWs was enhanced as the number of days engaged in caring for patients increased. This is the first study to investigate the relation between stress and the number of days worked by frontline Japanese HCWs working either exclusively or temporarily in wards specifically for COVID-19 patients.

### Comparison of TMDP and the general psychological distress (K6) scores

This study found that temporary support staff had lower psychological distress than workers assigned exclusively to a COVID-19 ward, which was as expected. Because the 30 days used in the present survey was the peak period of the COVID-19 outbreak, the regular staff had a higher actual workload and did more overtime than the temporary support staff because they were providing care for COVID-19 on all working days. Prior research in Japan on the relation between workload and general psychological distress has shown that a too few holidays have an adverse impact on depressive symptoms [11]. Also, our previous report showed a positive correlation between increased workload and K6 in Japanese healthcare workers during the first wave of the COVID-19 epidemic [6].

No significant differences were found between the two groups in terms of TMDP and its subscales. The psychological subscale of the TMDP was found to be equivalent for both groups, indicating that COVID-19-related psychosocial stress does not depend on if a HCW works exclusively in a COVID-19 ward or as temporary support staff.

### Association between TMDP and general psychological distress

In the present study, TMDP total scores showed a positive correlation with K6. However, no correlation was found between TMDP and general psychological distress in group A. This indicates that psychosocial stress related to working with COVID-19 is associated with general psychological distress in the early stages of working in a COVID-19 ward. This is an important finding concerning COVID-19-related stress care for HCWs working with COVID-19 patients, suggesting that interventions for psychological and social stress are important, even during short periods of engagement.

### Correlation between days engaged in patient care and symptoms

Our results show that social stress specific to HCWs is enhanced when temporary support staff are engaged in patient care for long periods of time. When the number of days of COVID-19 care increases, temporary support staff need to be given special attention to ameliorate their COVID-19-related social stress.

**Fig. 3:**
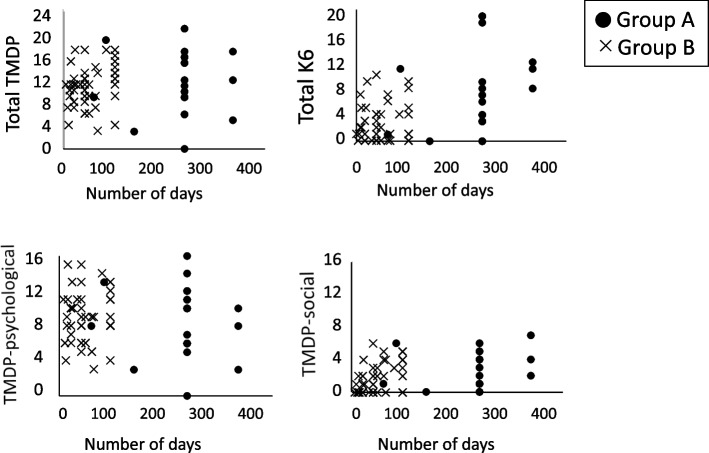
Correlations between the number of days engaged in patient care and the TMDP and K6 scores. Group A worked exclusively in Covid-19 wards and Group B temporarily

The reason there was no correlation between the number of days of care and any of the TMDP scales or the total score for group A may be that, according to the correlations shown in Fig. 3, there was no variation in the number of days of care for these workers. Also, such workers with relatively high number of days of care did not show high values, but remained at a constant value. Similarly, for K6 the distribution of days for group A workers was skewed, and participants with large number of working days did not necessarily show higher values, indicating a ceiling effect. These results will need to be clarified in future study that includes a more varied number of response days.

### Limitation

Most importantly, our measurement tools may be too few to adequately examine all of the important psychosocial symptoms among HCWs providing care for COVID-19 patients. Research using comprehensive and reliable questionnaires that include measures of workload and somatic symptoms are needed.

## Conclusion

Although temporary support staff experienced lower psychological distress than staff members working exclusively in COVID-19 wards, the psychosocial stress specific to COVID-19 care was comparable. Among the temporary support staff members, the general psychological distress of may be related to psychosocial stress specific to healthcare professionals during this COVID-19 outbreak, and the social stress was enhanced as the number of working days increased. Both staff members working exclusively and temporary support staff members should be provided with care focusing specifically on their COVID-19-related psychosocial stress. Future studies using more reliable and comprehensive assessments of stress and psychosomatic symptoms are desirable.

## Supplementary Information

**Additional file 1: Supplementary material 1.** Tokyo Metropolitan Distress Scale for Pandemic. **Supplementary Material 2.** The questionnaire items on the 6-item Kessler Scale.

## Data Availability

The datasets generated and/or analysed during the current study are not publicly available due to privacy reasons but are available from the corresponding author on reasonable request.

## Abbreviations

COVID-19: Coronavirus disease 2019
K6: The 6-item Kessler Scale
