# Supplementary material for: Differences in the presentation of COVID-19-related psychosocial stress and general psychological distress and the relation between the number of care days and these symptoms among Japanese ward staff working exclusively with COVID-19 and support staff
Source: Biopsychosoc Med. 2023 Apr 28;17:17. doi: 10.1186/s13030-023-00272-7 (PMC10147353; doi:10.1186/s13030-023-00272-7)
Supplement: Supplementary file 1 — Additional file 1: Supplementary material 1. Tokyo Metropolitan Distress Scale for Pandemic. Supplementary Material 2. The questionnaire items on the 6-item Kessler Scale. [file 13030_2023_272_MOESM1_ESM.docx]

Supplementary material 1

Tokyo Metropolitan Distress Scale for Pandemic (TMDP)

| During the PAST TWO WEEKS:  How frequently were you concerned about the following? | Never | Rarely | Occasionally | Often | Most of the time |
| --- | --- | --- | --- | --- | --- |
| 1. COVID-19 infection of oneself | 0 | 1 | 2 | 3 | 4 |
| 1. You have no control over whether you have COVID-19 or not | 0 | 1 | 2 | 3 | 4 |
| 1. Risk of COVID-19 patient care is unacceptable | 0 | 1 | 2 | 3 | 4 |
| 1. Safety of the work environment is not maintained in order to avoid being affected by COVID-19 | 0 | 1 | 2 | 3 | 4 |
| 1. Transmitting COVID-19 to people around you | 0 | 1 | 2 | 3 | 4 |
| 1. People around you avoid you because of your occupation | 0 | 1 | 2 | 3 | 4 |
| 1. Deteriorating workplace relationships in relation to COVID-19 | 0 | 1 | 2 | 3 | 4 |
| 1. Deteriorating family relationships in relation to COVID-19 | 0 | 1 | 2 | 3 | 4 |
| 1. Financial burden associated with COVID-19 | 0 | 1 | 2 | 3 | 4 |

Modified from Shiwaku H, et al. Novel brief screening scale, Tokyo Metropolitan Distress Scale for Pandemic (TMDP), for assessing mental and social stress of medical personnel in COVID-19 pandemic. Psychiatry Clin Neurosci. 2021;75:24-25. /CC BY-NC 4.0

Supplementary Material 2.

The questionnaire items on the 6-item Kessler Scale (K6).

| ***Q. During the last 30 days, about how often did…*** | |  |  |  |  |  |
| --- | --- | --- | --- | --- | --- | --- |
|  | | ***All*** | ***Most*** | ***Some*** | ***A little*** | ***None*** |
|  | | ***of the*** | ***of the*** | ***of the*** | ***of the*** | ***of the*** |
|  |  | ***time*** | ***time*** | ***time*** | ***time*** | ***time*** |
| …you feel nervous? | | 4 | 3 | 2 | 1 | 0 |
| …you feel hopeless? | | 4 | 3 | 2 | 1 | 0 |
| …you feel restless or fidgety? | | 4 | 3 | 2 | 1 | 0 |
| …you feel so depressed that nothing could cheer you up? | | 4 | 3 | 2 | 1 | 0 |
| …you feel that everything was an effort? | | 4 | 3 | 2 | 1 | 0 |
| …you feel worthless? | | 4 | 3 | 2 | 1 | 0 |

Modified from Greaves LM, Milojev P, Huang Y, Stronge S, Osborne D, et al. (2015) Regional Differences in the Psychological Recovery of Christchurch Residents Following the 2010/2011 Earthquakes: A Longitudinal Study. PLOS ONE 10(5): e0124278. <https://doi.org/10.1371/journal.pone.0124278> /CC BY 4.0
